# Molecular Docking of Bacterial Protein Modulators and Pharmacotherapeutics of *Carica papaya* Leaves as a Promising Therapy for Sepsis: Synchronising In Silico and In Vitro Studies

**DOI:** 10.3390/molecules28020574

**Published:** 2023-01-06

**Authors:** Juveria Usmani, Hina Kausar, Saleem Akbar, Ali Sartaj, Showkat R. Mir, Mohammed Jaseem Hassan, Manju Sharma, Razi Ahmad, Summaya Rashid, Mohd Nazam Ansari

**Affiliations:** 1 Department of Pharmacology, School of Pharmaceutical Education and Research, Jamia Hamdard University, New Delhi 110062, India; 2 Department of Pharmacognosy and Phytochemistry, School of Pharmaceutical Education and Research, Jamia Hamdard University, New Delhi 110062, India; 3 Department of Pharmaceutical Chemistry, School of Pharmaceutical Education and Research, Jamia Hamdard University, New Delhi 110062, India; 4 Department of Pharmaceutics, School of Pharmaceutical Education and Research, Jamia Hamdard University, New Delhi 110062, India; 5 Department of Pathology, Jawaharlal Nehru Medical College, Aligarh Muslim University, Aligarh 202002, India; 6 Department of Pharmacology, Hamdard Institute of Medical Sciences and Research, Jamia Hamdard University, New Delhi 110062, India; 7 Department of Pharmacology & Toxicology, College of Pharmacy, Prince Sattam Bin Abdulaziz University, Al-Kharj 11942, Saudi Arabia

**Keywords:** *Carica papaya*, in silico, cytotoxicity, ethanol extract, sepsis

## Abstract

Sepsis is a serious health concern globally, which necessitates understanding the root cause of infection for the prevention of proliferation inside the host’s body. Phytochemicals present in plants exhibit antibacterial and anti-proliferative properties stipulated for sepsis treatment. The aim of the study was to determine the potential role of *Carica papaya* leaf extract for sepsis treatment in silico and in vitro. We selected two phytochemical compounds, carpaine and quercetin, and docked them with bacterial proteins, heat shock protein (PDB ID: 4PO2), surfactant protein D (PDB ID: 1PW9), and lactobacillus bacterial protein (PDB ID: 4MKS) against imipenem and cyclophosphamide. Quercetin showed the strongest interaction with 1PW9 and 4MKS proteins. The leaves were extracted using ethanol, methanol, and water through Soxhlet extraction. Total flavonoid content, DPPH assay, HPTLC, and FTIR were performed. In vitro cytotoxicity of ethanol extract was screened via MTT assay on the J774 cell line. Ethanol extract (EE) possessed the maximum number of phytocomponents, the highest amount of flavonoid content, and the maximum antioxidant activity compared to other extracts. FTIR analysis confirmed the presence of N-H, O-H, C-H, C=O, C=C, and C-Cl functional groups in ethanol extract. Cell viability was highest (100%) at 25 µg/mL of EE. The present study demonstrated that the papaya leaves possessed antibacterial and cytotoxic activity against sepsis infection.

## 1. Introduction

Plants have been believed to be beneficial for humans since the days of yore. Traditional medicines and their active constituents are counted as potent sources of remedies for various ailments. Active ingredients in plant extracts like alkaloids, tannins, flavonoids, saponins, enzymes, volatile oils, etc., possess significant pharmacological activities [1]. *Carica papaya* is one such plant, popularly consumed around the world with the likelihood to provide numerous medicinal and nutraceutical benefits [2,3]. The papaya plant belongs to the family *Caricaceae*, is popularly cultivated in tropical and sub-tropical areas, and has been used historically in traditional medicine to cure and manage various disease conditions [4]. Different parts of the papaya plant such as its leaves, barks, roots, latex, fruit, flowers, and seeds are used and have considerable therapeutic benefits, including restoration of damaged skin, digestion of food, removal of dandruff, and pain relief [1,5]. Previous studies have reported anti-hypertensive, anthelminthic, antibacterial, diuretic, anti-fertility, hypolipidemic, antifungal, antitumor, antithrombocytopenic, and platelet-enhancing effects of *C. papaya* [6,7,8,9,10,11]. Studies on the extract of *C. papaya* have also shown immunomodulatory and anti-inflammatory potentials by inhibiting release of pro-inflammatory and anti-inflammatory cytokines [12]. Papaya leaves have also been reported to possess antioxidant properties and platelet-enhancing activity along with antithrombocytopenic properties [9,10]. In silico molecular docking is known to be one of the most efficient ways to reduce financial stress in research. Molecular docking is a useful tool for drug discovery campaigns, especially for virtual screening, and has many limitations. These molecular data provide insight into the formation of a ligand–receptor complex and its binding affinity [13]. According to a previous study, heat shock protein has exhibited a cytoprotective effect against stressful conditions, including inflammation, tissue injury, and oxidative stress [14]. Additionally, surfactant protein (PDB ID: 1PW9) is antimicrobial in nature and potentially inhibits cell proliferation of gram-negative bacteria by the action of improving cell membrane permeability [15]. Lactobacillus bacterial protein (PDB ID: 4MKS) was found to be the primary cause of septic urinary infection [16]. According to a study, 0.04 mg/g of quercetin was present in 0.25 mg/g of methanolic extract of papaya leaves [17]. Quercetin had the potential to inhibit the proliferation and activation of macrophages and cytokine stimulation induced by LPS, which allowed for the use of quercetin for the treatment of inflammatory bowel disease [18]. Quercetin exhibited antibacterial effects by significantly decreasing the presence of *Pseudomonas aeruginosa*, *Salmonella enterica*, *Staphylococcus aureus*, and *E. coli* [19]. On the other hand, carpaine contained in papaya leaves is responsible for antitumor, anticancer, and antimicrobial properties. The secondary metabolite contents of the papaya leaves, namely, carpaine alkaloids and pseudocarpaineam, are in the piperidine group of alkaloids. The piperidine-type alkaloid compounds have anticancer activity by virtue of inducing apoptosis [20].

Thus, the development of papaya leaf medications is needed through several in silico and in vitro studies.

Thin-layer chromatography is the simplest and most low-cost technique among other chromatographic methods [21]. High-performance thin-layer chromatography (HPTLC) has become a rational and important analytical option that is a simple, sophisticated, more powerful, and effective tool that works on the principle of the detection, separation, and authentication of metabolites and their products [22]. Therefore, the present study utilizes this technique for the detection, separation, and authentication of ethanol extract from *C. papaya* leaves.

In the past, studies have shown the cytotoxic effects of papaya leaves on several cell lines, demonstrating reduced cell viability in vitro [23]. In a study by Joseph et al., methanol extract from papaya leaves was found to be cytotoxic, whereas chloroform extract from the same plant was non-cytotoxic [24]. This demonstrates that the cytotoxic activity of a plant extract corresponds to the presence of different phytoconstituents. Despite the preceding cytotoxic studies on papaya leaves, there seems to be a paucity of information on the cytotoxic effect of ethanol extract from papaya leaves on the sepsis cell line. MTT assay is a widely used standard colorimetric assay to evaluate the cell viability of all types of cells in the culture media [25,26]. Considering the capabilities of the papaya plant, the objective of this study is to systematically evaluate the phytoconstituents present in various extracts of *C. papaya* leaves and explore their cytotoxic effect on a sepsis cell line, providing comprehensive insights that can be leveraged for therapeutic application in future. 

## 2. Results

### 2.1. Molecular Docking In Silico Study

**Figure 1:**
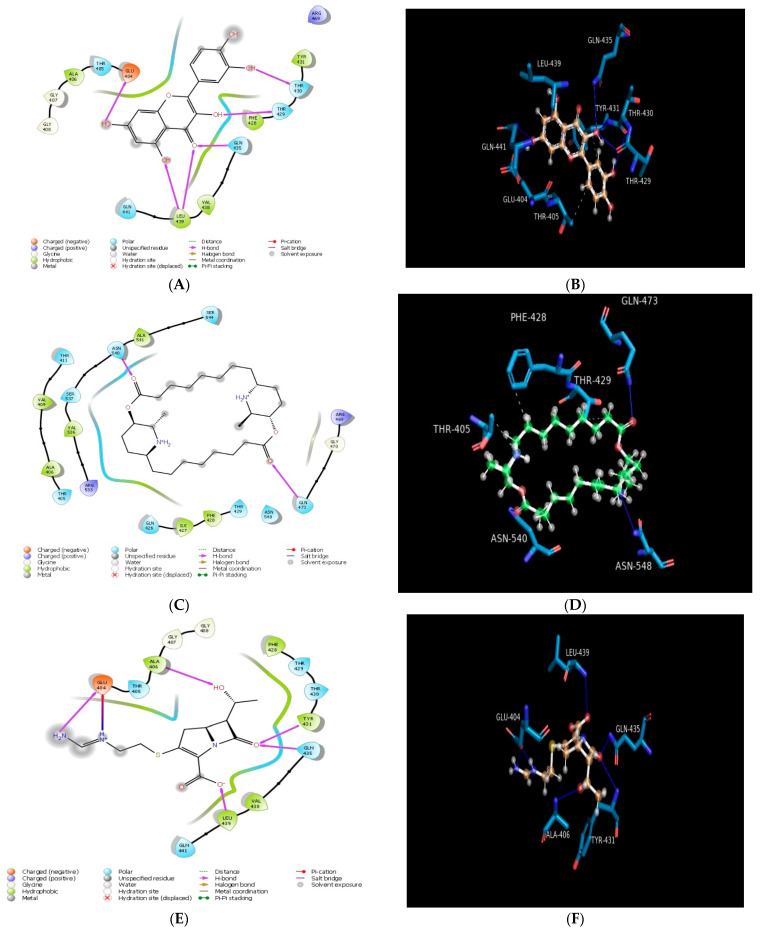

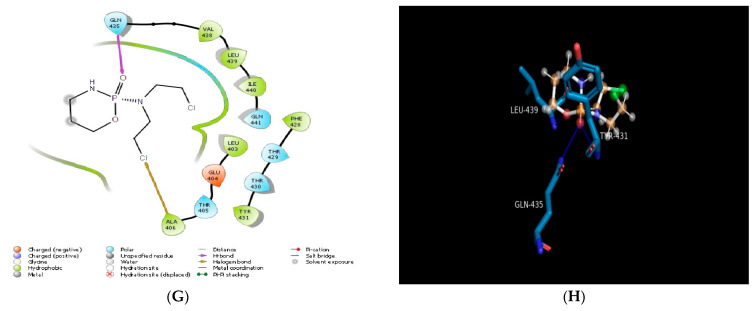
Molecular docking of compounds with heat shock protein (PDB ID: 4PO2): (**A**) 2D schematic diagram showing interactions of quercetin. (**B**) Cartoon view of heat shock protein with quercetin. (**C**) 2D schematic diagram showing interactions of carpaine. (**D**) Cartoon view of heat shock protein with carpaine. (**E**) 2D schematic diagram showing interactions of imipenem (standard). (**F**) Cartoon view of heat shock protein with imipenem (standard). (**G**) 2D schematic diagram showing interactions of cyclophosphamide (standard). (**H**) Cartoon view of heat shock protein with cyclophosphamide (standard). Residues involved in hydrogen bonding, van der Waals interactions, carbon–hydrogen, and pi–alkyl are represented in different colours indicated in the inset.

**Figure 2:**
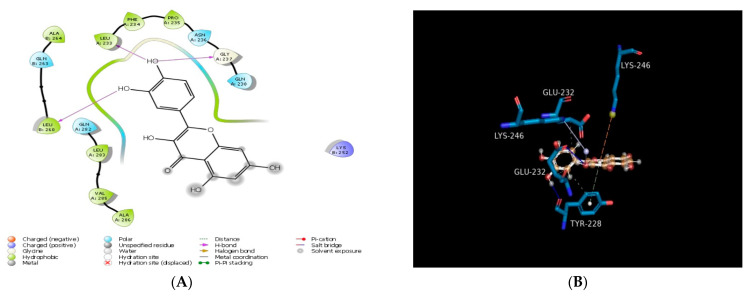

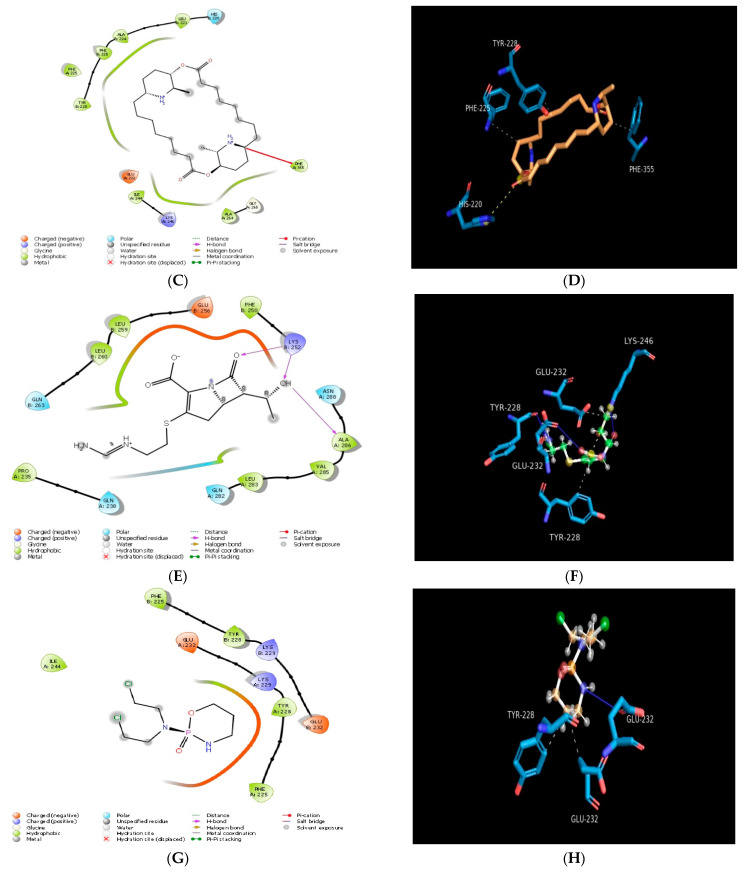
Molecular docking of compounds with surfactant protein D (PDB ID: 1PW9): (**A**) 2D schematic diagram showing interactions of quercetin. (**B**) Cartoon view of surfactant protein D with quercetin. (**C**) 2D schematic diagram showing interactions of carpaine. (**D**) Cartoon view of surfactant protein D with carpaine. (**E**) 2D schematic diagram showing interactions of imipenem (standard). (**F**) Cartoon view of surfactant protein D with imipenem (standard). (**G**) 2D schematic diagram showing interactions of cyclophosphamide (standard). (**H**) Cartoon view of surfactant protein D with cyclophosphamide (standard). Residues involved in hydrogen bonding, van der Waals interactions, carbon–hydrogen, and Pi–alkyl are represented in different colours indicated in the inset.

Molecular docking was performed in order to establish the binding ability of carpaine, quercetin, imipenem, and cyclophosphamide with heat shock protein, surfactant protein D, and lactobacillus bacterial protein. The docking scores of all compounds are presented in Table 1. All the compounds were found to exhibit several molecular interactions (hydrogen bond, pi–pi interaction, and hydrophobic interaction) with the target protein and were considered to be responsible for the antibacterial activity of the compounds. Among all the titled compounds tested for antibacterial activity, quercetin (test compound) was found to be most potent against two proteins, heat shock protein (PDB ID: 4PO2) and lactobacillus bacterial protein (PDB ID: 4MKS), and have the highest docking score (−6.04 and −5.86 Kcal/mol) as compared to imipenem and cyclophosphamide. Quercetin also exhibited a weak docking score against surfactant protein D (PDB ID:1PW9) with a comparable docking score (−4.48 Kcal/mol) (Table 1). Quercetin demonstrated a hydrogen bond with amino acid residues (GLU404, LEU439, GLN435, THR429, THR430; 1.985 Å) as well as pi–pi stacking with amino acid residue (TYR 228) against heat shock protein (PDB ID: 4PO2) (Figure 1**).** Carpaine also assumes a favourable orientation within the binding site by interacting with other residues (GLN473, ASN540) as shown in Figure 1. The standard compound (imipenem) revealed several hydrogen bonds with amino acid residues (GLU404, ALA406, TYR431, GLN435, and LEU439) and also had a good docking score (−6.64 Kcal/mol) against heat shock protein (PDB ID: 4PO2) (Table 1 and Figure 1). It also exhibits interaction with amino acid residues (THR405, GLY407, GLY408, PHE428, THR429, THR430, VAL438, and GLN441). The standard compound (cyclophosphamide) showed a hydrophobic interaction with the amino acid residue GLN435. It also resulted in a weak docking score (−4.97 Kcal/mol) and interaction with some other amino acid residues (VAL438, LEU439, ILE440, GLN441, LEU403, GLU404, THR405, ALA406, PHE428, THR429, THR430, and TYR431) against heat shock protein (PDB ID: 4PO2) (Table 1). Another test compound (carpaine) demonstrated weak interaction with the amino acid residues (HIS220, LEU221, ALA224, PHE225, TYR228, GLU232, ILE244, LYS246, ALA264, GLY265, and PHE355) and has the lowest docking score (−2.71 Kcal/mol) against surfactant protein D (PDB ID: 1PW9) (Table 1 and Figure 2). A molecular docking simulation was performed for the titled compounds and displayed good MMGBSA binding energies in the range of −51.31 to −11.03 Kcal/mol (Table 1). The test compounds carpaine and quercetin presented −24.65 and −40.54 Kcal/mol, respectively, binding free energies against surfactant protein D (PDB ID: 1PW9), −41.41 and −38.38 Kcal/mol, respectively, against heat shock protein (PDB ID: 4PO2), and −32.87 and −38.71 Kcal/mol, respectively, against lactobacillus bacterial protein (PDB ID: 4MKS). The redocking of a docked complex of standard (cyclophosphamide) with heat shock protein (PDB ID: 4PO2), surfactant protein D (PDB ID: 1PW9), and lactobacillus bacterial protein (PDB ID: 4MKS) exhibited a similar docking mode with RMSD values of 0.0598, 0.0172, and 0.000 Å, respectively. The proteins namely, surfactant protein D (PDB ID: 1PW9), heat shock protein (PDB ID: 4PO2), and lactobacillus bacterial protein (PDB ID: 4MKS) and ZINC00630526 was the only compound that passed the virtual screening, and the docking score of ZINC00630526 were −2.309 (1PW9), −2.127 (heat shock protein (PDB ID: 4PO2)), and −2.406 (lactobacillus bacterial protein (PDB ID 4MKS)), respectively, which are very low compared to known inhibitors (imipenem), i.e., −4.20 (surfactant protein D (PDB ID: 1PW9)), −6.64 (heat shock protein (PDB ID: 4PO2)), and −5.34 (lactobacillus bacterial protein (PDB ID: 4MKS)). After completing the experiment, the known inhibitors (imipenem) ranked one, one, one against surfactant protein D (PDB ID: 1PW9), heat shock protein (PDB ID: 4PO2), and lactobacillus bacterial protein (PDB ID: 4MKS), respectively, among the randomly selected non-inhibitor molecules.

**Figure 3:**
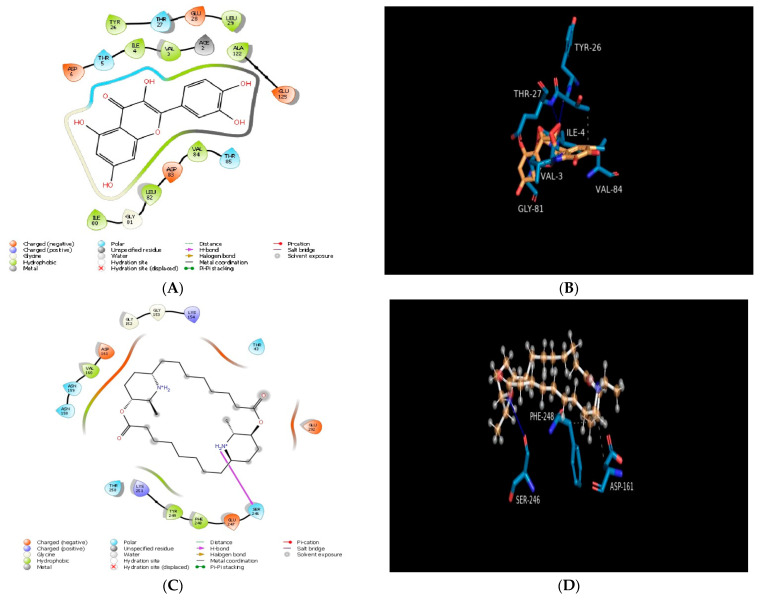

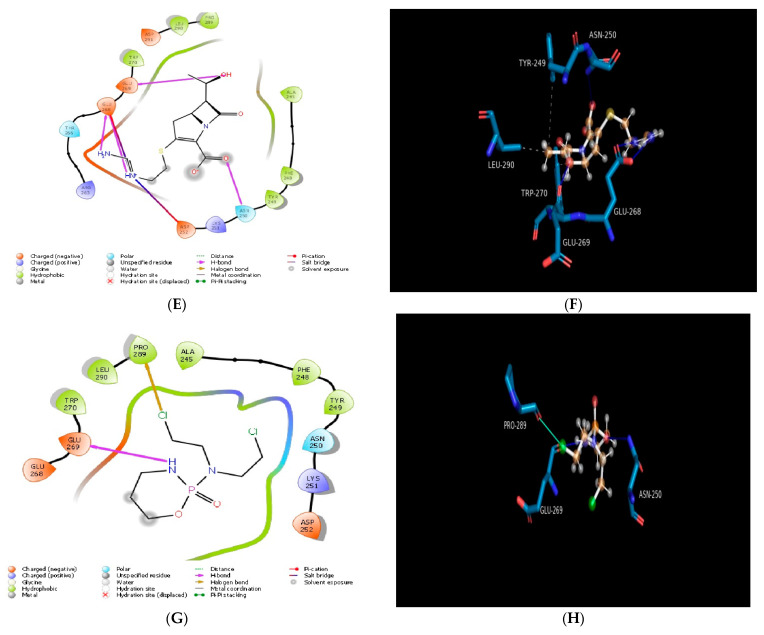
Molecular docking of compounds with lactobacillus bacterial protein (PDB ID: 4MKS): (**A**) 2D schematic diagram showing interactions of quercetin. (**B**) Cartoon view of lactobacillus bacterial protein with quercetin. (**C**) 2D schematic diagram showing interactions of carpaine. (**D**) Cartoon view of lactobacillus bacterial protein with carpaine. (**E**) 2D schematic diagram showing interactions of imipenem (standard). (**F**) Cartoon view of lactobacillus bacterial protein with imipenem (standard). (**G**) 2D schematic diagram showing interactions of cyclophosphamide (standard). (**H**) Cartoon view of lactobacillus bacterial protein with cyclophosphamide (standard). Residues involved in hydrogen bonding, van der Waals interactions, carbon–hydrogen, and pi–alkyl are represented in different colours indicated in the inset.

We screened test and reference compounds against lactobacillus bacterial protein (4MKS). After screening, we found that quercetin demonstrated very good results, with a docking score value of −5.86 Kcal/mol, and showed hydrophobic interaction with the amino acid residues (VAL3, ILE4, TYR26, LEU29, ILE80, GLY81, LEU82, VAL84, and ALA122) (Table 1). On the other hand, carpaine exhibited one hydrogen bond with the amino acid residue SER246 and had a lower docking score of −4.36 Kcal/mol compared to quercetin and imipenem, but a higher docking score compared to cyclophosphamide. The 2D and 3D interaction diagram of quercetin, carpaine, imipenem, and cyclophosphamide are represented in Figure 3.

**Figure 4:**
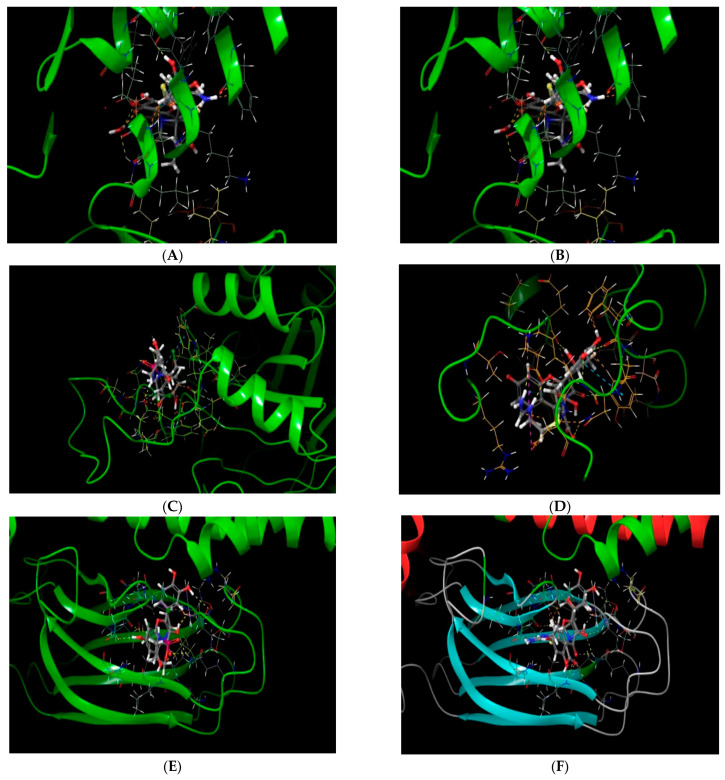
(**A**) Superimposing image of quercetin and cyclophosphamide at the active binding site of surfactant protein D (PDB ID: 1PW9). (**B**) Superimposing image of quercetin and imipenem at the active binding site of surfactant protein D (PDB ID: 1PW9). (**C**) Superimposing image of quercetin and cyclophosphamide at the active binding site of lactobacillus bacterial protein (4MKS). (**D**) Superimposing image of quercetin and imipenem at the active binding site of lactobacillus bacterial protein (4MKS). (**E**) Superimposing image of quercetin and cyclophosphamide at the active binding site of heat shock protein (PDB ID: 4PO2). (**F**) Superimposing image of quercetin and imipenem at the active binding site of heat shock protein (PDB ID: 4PO2).

Among the tested compound, quercetin demonstrated the best interaction with heat shock protein (PDB ID: 4PO2) and lactobacillus bacterial protein (4MKS) as compared to surfactant protein D (PDB ID: 1PW9) and bound to the active binding domain of all three proteins as shown in the superimposed image of Figure 4.

### 2.2. Percentage Yield

The percentage yield of ethanol, methanol, and aqueous extracts of papaya leaves was calculated using a standard formula and found to be 13.1%, 12.2%, and 10.6%, respectively. The percentage yield of ethanol extract was the highest among all three extracts.

### 2.3. Phytochemical Screening of Extracts

The qualitative analysis of different extracts of papaya leaves indicates the presence of various phytoconstituents, as shown in Table 2. Ethanolic extract was found to contain a higher number of phytoconstituents as compared to aqueous and methanolic extract. Alkaloids, flavonoids, terpenoids, saponins, and glycosides were found in ethanol extract. Only alkaloids, flavonoids, and glycosides were present in the aqueous extract, whereas methanol extract was found to contain alkaloids, flavonoids, phenolic compounds, and saponins in trace amounts. Therefore, we proceeded with further analysis of the ethanol extract.

### 2.4. Estimation of Total Flavonoids

The total flavonoid content was found to be 1.83 ± 0.16%, 1.16 ± 0.28%, and 2.23 ± 0.24% for aqueous, methanol, and ethanol extracts, respectively, of *Carica papaya* leaves. The given values are expressed as mean ± SD of three different determinations.

### 2.5. DPPH Radical Scavenging Activity

The examination of the antioxidant activity of different extracts of papaya leaves was carried out using ascorbic acid as standard in DPPH assays as presented in Table 3.

**Figure 5:**
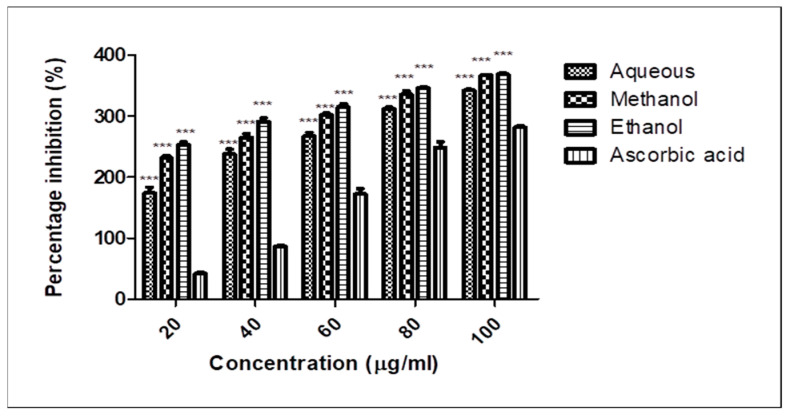
DPPH free radical scavenging activity of different extracts of *C. papaya* leaves. Different solvents like methanol, aqueous, and ethanol were used to obtain extracts of papaya leaves, which underwent DPPH assay at different concentrations to determine the free radical scavenging activity of these extracts and compare with ascorbic acid as standard antioxidant. A significant difference (*** *p* < 0.001) was observed between these extracts and the standard. The methanol and ethanol extracts showed higher significance, while the aqueous extract displayed comparatively less significant values indicative of antioxidant characteristics.

Data from multiple groups of treatment were analysed by two-way ANOVA using the Bonferroni post-test. Values are expressed as mean ± SD (n = 3). A significant variation was observed in the three extracts of leaves as compared to ascorbic acid (Figure 5).

A significant variation was observed in the three extracts of leaves as compared to ascorbic acid (Figure 5). The results demonstrated dose-dependent free radical scavenging activity at a concentration of 20–100 mg/mL. The free radical scavenging activity of the aqueous, methanol, and ethanol extracts was found to be 51.26 ± 1.47–80.66 ± 0.09, 70.43 ± 3.47–89 ± 1.2, and 77.86 ± 3.08–89.63 ± 1.73 units, respectively, each of which was significantly higher than that of ascorbic acid extract, which was 7.23 ± 1.67–60.76 ± 2.28. Data from multiple groups of treatment were analysed by two-way ANOVA using the Bonferroni post-test. A statistically significant difference was expressed as *** *p* < 0.001 between the groups.

### 2.6. TLC Analysis

**Figure 6:**
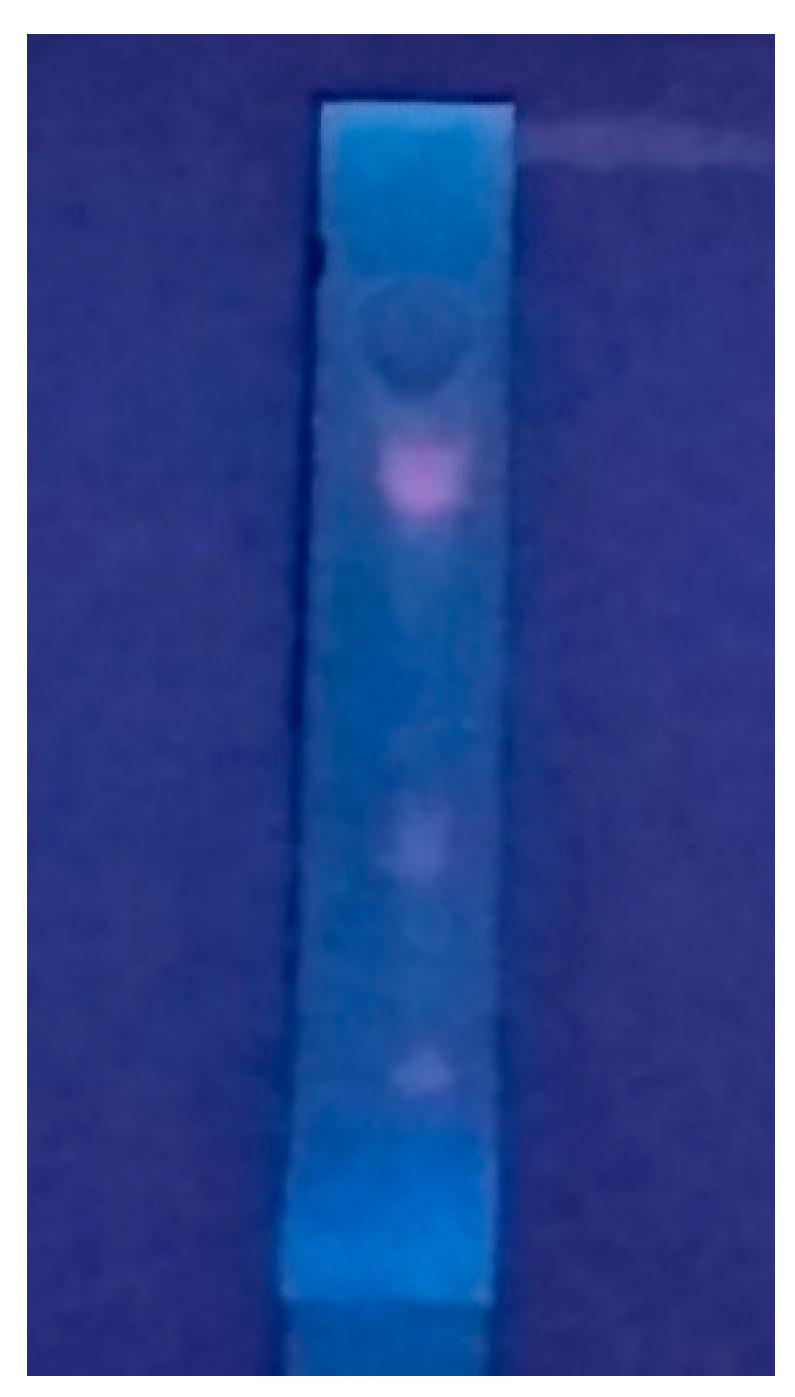
TLC plate observed in UV chamber.

TLC is one of the easiest and most common techniques based on the principle of identification and separation of various phytocomponents in an herbal drug. In our study, TLC was performed for ethanolic extract of papaya leaves where three spots were observed in the UV region as shown in Figure 6.

### 2.7. HPTLC Fingerprinting

**Figure 7:**
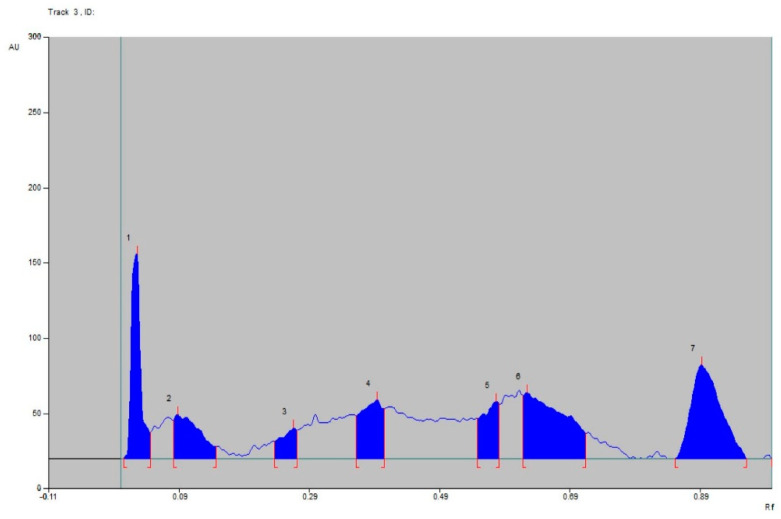
Chromatogram of ethanol extract of *C. papaya*.

High-performance thin-layer chromatography (HPTLC) fingerprinting was carried out using toluene: ethylacetate: formic acid (8:1.5:0.5) as the mobile phase for the purpose of identification of phytochemical constituents in the papaya leaf extract. Peaks were observed at 254 nm with different Rf values in the HPTLC chromatogram (Figure 7). A total of 7 peaks with Rf values 0.05, 0.15, 0.27, 0.40, 0.58, 0.71, and 0.96 were obtained at 254 nm.

### 2.8. FTIR Spectrum

**Figure 8:**
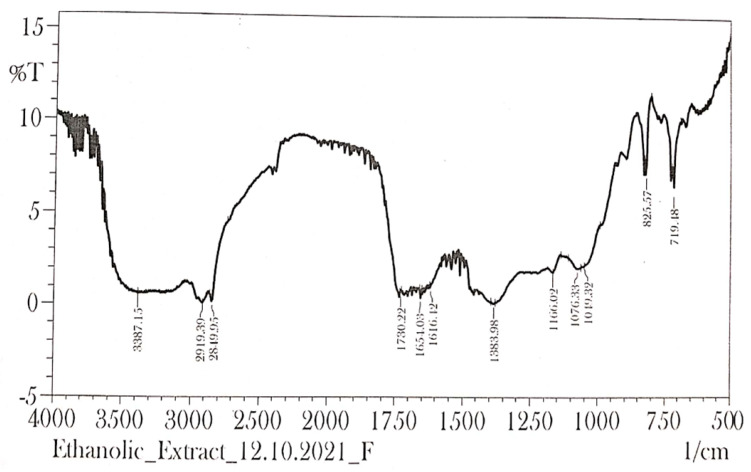
FTIR spectrum of ethanol extract of *Carica papaya* leaves.

As seen in Figure 8, the FTIR analysed the presence of different kinds of molecules in the papaya extract. Peaks observed at 3387.15/cm correspond to N-H stretch (amines) and O-H stretch (alcohols); peaks observed at 2919.39/cm relate to C-H stretch (alkanes) and O-H stretch (carboxylic acids); peaks observed at 2819.95/cm are C-H stretch (aldehydes) and O-H stretch (carboxylic acids); peaks observed at 1730.22/cm are associated with C=O stretch (aldehydes) and C=O stretch (ketones); peaks observed at 1651.03/cm are related to C=O stretch (amides) and C=C stretch (alkenes); peaks observed at 1616.12/cm are C=C stretch (alkenes) and C=C stretch (aromatic rings); while peaks observed at 825.57 and 719.18 are associated with =C-H bend (alkenes), C-H bend (aromatic compounds), and C-Cl stretch (alkyl and aryl halides).

### 2.9. In Vitro % Cell Viability

**Figure 9:**
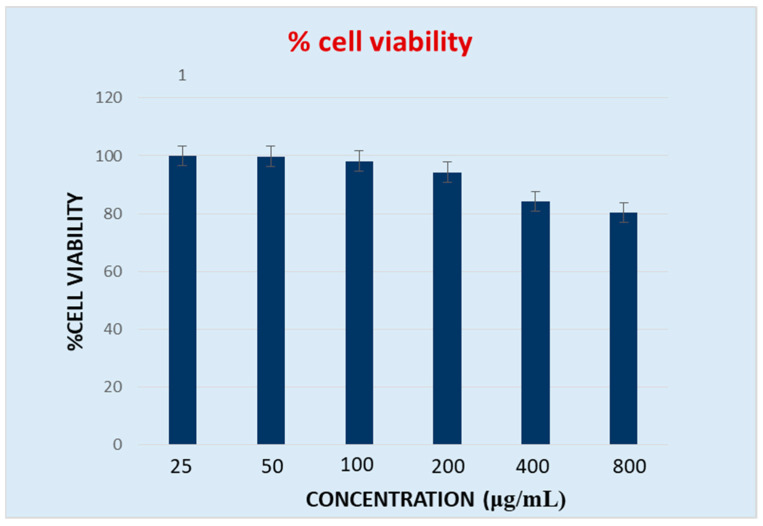
Effect of ethanol extract of *C. papaya* leaves on percentage viability of J774 cells.

As represented in Figure 9, the percentage cell viability of J774 cells upon treatment with increasing concentrations (25, 50, 100, 200, 400, and 800 µg/mL) of ethanol extract of papaya leaves was found to be 100%, 99.8%, 98.2%, 94.3%, 84.2%, and 80.3%, respectively. Cell viability was found to be highest (100%) at 25 µg/mL and lowest (80.3%) at 800 µg/mL of ethanol extract (EE), indicating that the lowest concentration of extract is unable to inhibit the total population of J774 cells, whereas the highest concentration of extract can inhibit around 20% of the cell population. This indicates that 800 µg/mL of extract produces the highest cytotoxicity.

## 3. Discussion

Infectious diseases have been a global burden and a major cause of death. Sepsis is an immunocompromised infection that occurs due to the host’s response to injury. Treatment of sepsis commonly relies on antibiotic therapy; however, due to the increasing incidence of antibiotic resistance, the treatment approach remains limited [27]. In silico antibacterial activity of *C. papaya* leaves was performed by using bioinformatics tools. The flavonoid quercetin exhibited the highest interaction with the bacterial protein. The present study is suggestive of the fact that several van der Waals, covalent, carbon–hydrogen, pi–alkyl, and electrostatic interactions were observed to be the key forces for bonding of quercetin, carpaine, imipenem, and cyclophosphamide together with the heat shock protein (PDB ID: 4PO2), surfactant protein D (PDB ID: 1PW9), and lactobacillus bacterial protein (PDB ID: 4MKS). According to a previous study, heat shock protein (PDB ID: 4PO2) has exhibited a cytoprotective effect against stressful conditions, including inflammation, tissue injury, and oxidative stress [14]. Also, surfactant protein (PDB ID: 1PW9) is antimicrobial in nature and potentially inhibits cell proliferation of gram-negative bacteria by the action of improving cell membrane permeability [15]. Another study reported that the enteral surfactant protein D worsens mortality after CLP by enhancing bacterial colonization in the gut [28]. Lactobacillus bacterial protein (PDB ID: 4MKS) was found to be the primary cause of septic urinary infection [16]. In conjunction with this, the results from our study have shown an excellent docking score by producing the interaction of quercetin and carpaine with the heat shock protein (PDB ID: 4PO2) and lactobacillus bacterial protein (PDB ID: 4MKS), but comparatively less binding with surfactant protein D (PDB ID: 1PW9), thus confirming the beneficial cytoprotective and antibacterial activity of the titled compounds for sepsis treatment. Moreover, this study indicated that *C. papaya* might employ antibacterial activity, which could be a platform to investigate the role of test compounds against sepsis.

*C. papaya* is enriched with antioxidants including α-tocopherol, ascorbic acid, various flavonoids, phenolic compounds, glycosides, enzymes, etc., and is commonly used for the prevention and treatment of innumerable diseases [1,29]. An important therapeutic application of papaya leaves has been its use as an antithrombocytopenic drug to treat dengue fever [10,30]. Phytochemical screening of papaya leaves extracts has identified alkaloids, terpenoids, flavonoids, saponins, steroids, tannins, and phenols that have shown potent therapeutic significance against inflammation, oxidative stress, and hypoglycaemic conditions [12,29,31]. The present study also confirms the presence of these chemical compounds in *C. papaya* leaves and suggests that the amount of the phytochemicals such as alkaloids, flavonoids, terpenoids, saponins, and glycosides was highest in ethanol extracts as compared to aqueous and methanol extracts that remained the mainstream of the present study (Table 1). The highest percentage yield of papaya leaves was 13.1% in ethanol extract.

Flavonoids are important phytocomponents that possess antioxidant and anti-inflammatory properties. Alkaloids are also widely distributed phytoconstituents sought after for their anti-inflammatory, antimalarial, stimulant, narcotic, analgesic, antispasmodic, and antitumoral properties [31]. Therefore, the current study can be valuable in further assessing quantitative parameters of phytotherapeutically active molecules. The total flavonoid content was highest in ethanol extract as compared to methanol and aqueous extracts, which correlates with previous studies [32,33]. These findings confirm that *C. papaya* leaves contain a significant quantity of flavonoid compounds, which exert an anti-inflammatory effect.

Several types of assays are being included as potent tools to quantify the antioxidant potential of natural products. The DPPH free radical scavenging assay is usually preferred over other methods due to its stability, simplicity, reproducibility, feasibility, and efficiency [34]. The present study has displayed the potent oxidative stress-reducing potential of *C. papaya* leaf extracts. In vitro, a DPPH assay was carried out to determine the antioxidant potential of papaya leaves, demonstrating higher significance in ethanol and methanol extracts and lesser in the aqueous extract as compared to ascorbic acid, which is conducive to inhibiting oxidative stress levels corresponding to previous studies [32]. Taking into account the positive outcomes of ethanol extract, it was selected for further analysis and examination of cytotoxicity using a sepsis cell line.

HPTLC has become a rational and important analytical option that is a simple, sophisticated, more powerful, and effective tool for the detection, separation, and authentication of herbal drugs and their products [22]. HPTLC fingerprinting of ethanolic extract of *C. papaya* leaves demonstrated the presence of various phytoconstituents in the ethanol extract. The chromatogram observed 7 peaks at different Rf values detected at 254 nm in the toluene: ethylacetate: formic acid (8:1.5:0.5) solvent system, indicating the number of constituents in the extract that can be further utilized to evaluate its therapeutic efficacy. FTIR is the most significant tool for the identification of functional groups present in phytomedicines [34]. The FTIR analysis indicated the presence of numerous characteristic functional groups such as: N-H, indicated as amines; O-H, indicating the presence of amines or carboxylic acid; C=O, specified as aldehydes, ketones, or amides; C=C, indicating the presence of alkenes; and the aromatic ring in ethanol extract of papaya leaves, holding prolific medicinal properties. The presence of saponins in *C. papaya* leaves has shown cytotoxic effects through increased cell permeability [35]. This directly correlates with our results, indicating the presence of saponins in the leaf extract of the papaya plant. It is thus, necessary to analyse the cytotoxicity of the plant leaves. MTT assay is a standard colorimetric assay widely used to evaluate the cell viability of all types of cells in the culture media. The measurement of cell viability in this method corresponds to cellular respiration and the quantity of formazan produced, indicating the number of viable cells in the culture incorporated with the test/standard agent. The presence of a higher number of viable cells in the culture results in higher levels of formazan crystal formation, which helps in determining cell proliferation and thus the cytotoxicity of the treatment used [25,36]. No evidence is available to date on the cytotoxic effect of EE on the J774 sepsis cell lines. Hence, in vitro cytotoxicity was carried out using an MTT assay at different concentrations of ethanol extract of papaya leaves. The results revealed that the cell viability was at a maximum (100%) at the lowest concentration (25 µg/mL) and lowest (80.3%) at the highest concentration (800 µg/mL), which confirms that the cytotoxic activity of the plant was observed at the highest concentration (800 µg/mL) of EE tested. The results showed that the EE inhibits cell proliferation and produces significant sepsis managing potentials. This further necessitates in-depth investigation on exploring the potential of papaya leaves for managing sepsis and related complications.

## 4. Materials and Methods

### 4.1. Sample Collection and Authentication

Fresh leaves of *C. papaya* were collected from the campus of Jamia Hamdard, and a voucher specimen (BOT/DAC/2021/06) was deposited in the herbarium of the Department of Botany, School of Chemical and Life Sciences, Jamia Hamdard, New Delhi, where identification of leaves was carried out by a taxonomist.

### 4.2. In Silico Study

The molecular docking study was carried out to establish different interactions between the test compounds and the target protein. The 3D structure of heat shock protein (PDB ID: 4PO2), surfactant protein D (PDB ID: 1PW9), and lactobacillus bacterial protein (4MKS) was performed on a Maestro 12.5 program (Schrodinger Inc., New York, NY, USA) using a the 64-bit operating system [Intel (R) Core (TM) i3-7020U CPU @ 2.30 GHz, 8 GB RAM]. The X-ray crystal structure, heat shock protein (PDB ID: 4PO2) [37], surfactant protein D (PDB ID: 1PW9) [38], and lactobacillus bacterial protein (PDB ID: 4MKS) [39] with the known inhibitor (imipenem and cyclophosphamide), was retrieved from the RCSB protein Data Bank (http://www.pdb.org/pdb/home/home.do accessed on 26 July 2022). The protein obtained was first prepared using the protein preparation wizard module. Water molecules and all other undesirable residues were removed, and hydrogen atoms were added before subjecting to the docking process. Site mapping was performed by selecting minimized proteins using the sitemap module of Schrodinger. We have selected a particular site for docking based on a high site score among the generated binding site.

The grid was prepared using minimized protein, which indicates the drugs have binding sites related to the specific target. The prepared grid was used for further processing in the advanced docking process. The grid box was generated according to the active site of the protein (4PO2, 1PW9, and 4MKS) where the centre was X: 50.0, Y: 25.0, Z: 70.0 (coordinates), X: 20.0, Y: 25.0, Z: 10.0 (coordinates), and X: −30.0, Y: −50.0, Z: 10.0 (coordinates), respectively. With no restrictions, the van der Waals radius scaling factor was set to 1.0. Finally, the grid was created with a partial charge cut-off of around 0.25. The amino acid residues SER400, THR405, THR411, SER418, THR422, THR425, THR429, THR430, TYR443, THR450, THR491, THR502, THR504, SER537, and SER544 for the protein (PDB ID: 4PO2); SER226, TYR228, SER239, THR247, and THR262 for the protein (PDB ID: 1PW9); and SER15, THR144, SER174, THR185, THR188, TYR231, CYS243, SER285, SER335, TYR361, THR362, SER366, THR372, THR384, THR391, SER393, THR395, TYR403, TYR419, SER424, and TYR426 for the protein (PDB ID: 4MKS) were considered for the grid generation. The Receptor Grid Generation tool in Maestro was used to generate the grid. ChemDraw 12.0 software (PerkinElmer Inc., Cambridge, MA, USA) was used to draw the structure of ligand molecules as a mol file. Their energy was minimized using the LigPrep module of Maestro. All possible ionization states at pH 7.0 ± 2.0 were generated and minimized. Ligand molecules prepared were docked into the active site in extra precision mode (XP) using Glide. Docking of carpaine and quercetin as test compounds and imipenem and cyclophosphamide as reference compounds was performed into the active site of each heat shock protein, surfactant protein D, and lactobacillus bacterial protein. The visualization was done to analyse the interaction between ligands and residues of amino acid residues on each protein by PyMOL software. The binding energy calculation (MM-GBSA) was further conducted using Prime in Maestro to analyse the potential biological response of the free binding energy of the ligands that are binding to the active site of the protein in the docked complex, using XP docking mode [40,41,42].

For validation of the docking protocol, redocking of docked compounds of the standard (cyclophosphamide) with heat shock protein (PDB ID: 4PO2), surfactant protein D (PDB ID: 1PW9), and lactobacillus bacterial protein (PDB ID: 4MKS) was performed. The RMSD measurements were calculated to determine the stability of the docking poses, which demonstrates the structural variation and protein stability. The decoy database was obtained from the DUD (a Directory of Useful Decoys), released on 22 October 2006 [43]. Fifty molecules (non-inhibitors) from the dataset and one known inhibitor (imipenem) were randomly selected. Further, virtual screening of the randomly selected non-inhibitors was performed against surfactant protein D (PDB ID: 1PW9), heat shock protein (PDB ID: 4PO2), and lactobacillus bacterial protein (PDB ID: 4MKS) using the virtual screening workflow module of Schrodinger, and docking scores were compared with the known inhibitors.

### 4.3. Preparation of Extracts

Fresh leaves obtained were washed thoroughly to remove dirt and impurities and air-dried for 2–3 days. These were then crushed and reduced to powdered form using a mortar pestle. Dried powdered leaves weighing 58 g were extracted with different solvents like ethanol, water, and methanol (500 mL) in the Soxhlet apparatus for 24 h. The temperature of the solvent was kept above 78 °C. The extract obtained was evaporated to dryness by a rotary evaporator at 65 °C and then kept in an oven at 64 °C until crude extract was obtained. The final extracts were stored at 2–4 °C until further estimations were made. The percentage yield of extract was calculated using the formula [44]:

$$\%\;\mathrm{yield}\;\mathrm{of}\;\mathrm{extract}=\frac{\mathrm{weight}\;\mathrm{of}\;\mathrm{extract}\;\mathrm{obtained}}{\mathrm{weight}\;\mathrm{of}\;\mathrm{powder}\;\mathrm{material}}\;\times \;100$$

### 4.4. Preliminary Estimation of Phytoconstituents

Analysis of phytoconstituents present in papaya leaves was made using water, methanol, and ethanol. Tests for chemical components like alkaloids, flavonoids, saponins, tannins, phenolic compounds, terpenoids, steroids, anthraquinones, cardiac glycosides, and volatile oils were performed following standard protocols from previous studies [45].

### 4.5. Total Flavonoid Content

The flavonoid content was determined by the method described in [46]. Ten milligrams of different extracts were weighed and mixed with respective solvents. The extracts were filtered with Whatman No. 42 filter paper. The filtrates were collected and evaporated to dryness on a water bath to a constant weight.

The flavonoid content was calculated using the following formula [32]:

$$\%\;\mathrm{flavonoids}=\frac{\mathrm{weight}\;\mathrm{of}\;\mathrm{final}\;\mathrm{filtrate}}{\mathrm{weight}\;\mathrm{of}\;\mathrm{sample}}\;\times \;100$$

### 4.6. DPPH Radical Scavenging Activity

Free radical scavenging activity of the sample extract was performed as described in a previous study [47]. DPPH is a commonly practiced assay for the evaluation of free radical scavenging potentials of the antioxidant content of pure compounds. This assay is considered a reliable, easy, and standard colorimetric method utilized for the characterization of antioxidant properties [48]. An amount of 0.004% *w*/*v* DPPH (2,2-diphenyl-l-picryl hydrazyl) solution was prepared in 95% methanol. 100 mL of stock solution of plant extracts in standard ascorbic acid was then prepared at a concentration of 100 µg/mL. From this stock solution, 2 mL, 4 mL, 6 mL, 8 mL, and 10 mL of this solution were mixed with methanol to make the final volume up to 10 mL, making the final concentration up to 20 µg/mL, 40 µg/mL, 60 µg/mL, 80 µg/mL, and 100 µg/mL, respectively. A freshly prepared DPPH solution (2 mL) was added to each test tube and left to be mixed in the dark for 15 min. Absorbance was measured at 523 nm against the blank using the UV spectrophotometer. For the control, the DPPH solution (2 mL) was mixed with methanol (10 mL). The assay was carried out in triplicate. DPPH free radical scavenging activity of the extracts was calculated as percentage inhibition (%) using the following formula:

$$\mathrm{DPPH}\;\mathrm{scavenging}\;\mathrm{activity}=\frac{\left[1-\{\mathrm{Abs}\;\mathrm{sample}-\mathrm{Abs}\;\mathrm{blank}\;\mathrm{sample})\right]}{\mathrm{Abs}\;\mathrm{control}}\;\times \;100$$

### 4.7. Thin Layer Chromatography Analysis

The ethanol extract of papaya leaves was analysed using thin-layer chromatography (TLC) to separate fractions of different active constituents of the drug. TLC plates (Merck-silica gel 60 F254) were developed to confirm the presence of fractions of different phytopharmaceuticals in the drug. It was allowed to run in a glass chamber containing a mobile phase. Different combinations of mobile phases were allowed to run through the drug sample to obtain the best visuals of separated constituents. The most appropriate outcome was obtained by the combination of toluene: ethylacetate: formic acid (8:1.5:0.5) solvent system. The plate was then observed to identify the positions of spots in an ultraviolet chamber at 254 nm. The Rf value of the herbal extract was calculated using the standard formula [49]:

$$\mathrm{Rf}=\frac{\mathrm{Distance}\;\mathrm{traveled}\;\mathrm{by}\;\mathrm{the}\;\mathrm{solute}}{\mathrm{Distance}\;\mathrm{traveled}\;\mathrm{by}\;\mathrm{the}\;\mathrm{solvent}\;}$$

### 4.8. HPTLC Fingerprinting Profile of Ethanolic Extract of Carica Papaya Leaves

High-performance thin-layer chromatography (HPTLC) fingerprinting was carried out using toluene: ethylacetate: formic acid (8:1.5:0.5) as a mobile phase for the purpose of identification of phytochemical constituents in the papaya leaf extract. Peaks were observed at 254 nm with different Rf values in the HPTLC chromatogram.

### 4.9. Fourier Transform Infrared Spectroscopy (FTIR)

FTIR is very economical, easy, fast, and requires a small amount of a sample. The functioning of the tool is based on the principle of identification of the presence of functional groups in the phytoconstituents [50,51]. A dried extract of ethanol extract of papaya leaves was taken for FTIR analysis. Pellets of leaf extract were prepared by mixing with 1–2 mg KBr powder to achieve a translucent powder, which was then compressed by the mechanical press to get the desired pellet. The spectrum was analysed by the means of IR solution software 3.50 build 214 (Shimadzu, Kyoto, Japan) to identify the presence of functional groups in the compound [52].

### 4.10. Cytotoxicity Study

The in vitro cell proliferation assay was performed using a 4,5-dimethylthiazole-2-yl)-2,5 diphenyltetrazolium bromide (MTT) assay. Briefly, J774 cells were seeded in a 96-well microplate. Cells were incubated at 37 °C until they attached to the bottom of wells. After 48 h of incubation, 40 µL RPMI (International PBI, Milan, Italy), taken as control, was removed, and cells were washed with 100 µL phosphate buffer. MTT (5 mg/mL) was subsequently added along with 1.6% DMSO as a positive control, and various concentrations of the EE (25, 50, 100, 200, 400, and 800 µg/mL) were added to 20 µL of J774 cell 106 suspensions (cells/mL). Imipenem as a positive control with different concentrations (0.25, 0.5, 1, 2, and 4 µg/mL) was added to other wells. The plates were incubated in a CO_2_ incubator at 37 °C for 24 hrs. The media in the plate was removed by aspiration. The absorbance was measured at 595 nm using a microplate reader (Microplate reader 680, Bio-Rad, Hercules, CA, USA) following the addition of 100 µL of distilled water, and the % cell viability was calculated using the following equation [53,54]:

$$\mathrm{Cell}\;\mathrm{viability}\;(\%)=\frac{\mathrm{OD}\;\mathrm{of}\;\mathrm{treated}-\mathrm{OD}\;\mathrm{of}\;\mathrm{control}}{\mathrm{OD}\;\mathrm{of}\;\mathrm{control}}\times 100$$

## 5. Conclusions

Several previous studies have demonstrated the antibacterial, antioxidant, anti-inflammatory, and cytotoxic activity of papaya leaves by inhibiting numerous pathways. Studies have reported the presence of the flavonoid quercetin to be responsible for the antioxidant, antibacterial, and anti-inflammatory properties. From our study, an excellent docking score of quercetin against surfactant protein D (PDB ID: 1PW9) and lactobacillus bacterial protein (PDB ID: 4MKS) was observed due to strong molecular interactions, such as hydrogen bonds, pi–pi interactions, and hydrophobic interactions, and favourable orientation within the binding site by interacting with other residues (GLU232, LYS229, TYR228, PHE225, VAL231, and CA404). This inhibits bacterial protein against infectious diseases, inflammation, and stressful conditions. Data also revealed that the papaya plant was a safe and effective therapeutic agent without possessing acute toxicity. Despite significant information available, including considerable in vitro cell line and in vivo studies, there is still a lack of evidence from clinical studies to determine the role of papaya leaves in the treatment of sepsis, as single-drug therapy, or as an adjuvant to immunomodulatory drugs. It is established by our study that *C. papaya* leaves have great antioxidant and cytotoxic potentials as identified by DPPH free radical scavenging activity and MTT assay. Additionally, the presence of flavonoids confers to the papaya leaves anti-inflammatory capabilities. Thus, ethanol extract of *Carica papaya* leaves can be a suitable candidate for future investigation as a potential herbal therapy for treating sepsis.

## Figures and Tables

**Table 1:** Detailed information on molecular docking of bacterial proteins with the test and standard compounds.

PDB ID	Compound	Docking Score	No. of Hydrogen Bond	Hydrogen Bond-Forming Residue	Another Interacting Residue	PRIME-MMGBSA Binding Free Energy (Kcal/mol)
1PW9	Carpaine	−2.71	n.f*	n.f*	TYR228, PHE225, ALA224, LEU221, HIS220, GLU232, ILE244, LYS246, ALA246, GLY265, PHE355	−24.65
	Quercetin	−4.48	02	TYR228	GLU232, LYS229, PHE225, TYR228, VAL231	−40.54
	Imipenem	−4.20	03	TYR228, LYS246	LEU233, GLU232, LYS229, PHE225, TYR228, VAL231, ILE244	−51.31
	Cyclophosphamide	−4.35	n.f*	n.f*	ILE244, GLU232, PHE225, TYR228, LYS229	−11.03
4PO2	Carpaine	−3.44	02	GLN473, ASN540	THR405, ALA406, VAL409, THR411, ARG533, VAL536, SER537, ASN540, ALA541, SER544, GLN426, ILE427, PHE428, THR429, ASN548, GLY470, ARG469	−41.41
	Quercetin	−6.04	05	GLU404, LEU439, GLN435, THR429, THR430	GLY408, GLY407, ALA406, THR405, GLU404, TYR431, PHE428, VAL438, GLN441	−38.38
	Imipenem	−6.64	05	GLU404, ALA406, TYR431, GLN435, LEU439	THR405, GLY407, GLY408, PHE428, THR429, THR430, VAL438, GLN441	−45.18
	Cyclophosphamide	−4.97	01	GLN435	VAL438, LEU439, ILE440, GLN441, LEU403, GLU404, THR405, ALA406, PHE428, THR429, THR430, TYR431	−39.28
4MKS	Carpaine	−4.36	01	SER246	ASN158, ASN159, VAL160, ASP161, GLY152, GLY153, LYS154, THR43, GLU292, GLU247, PHE248, TYR249, LYS251, THR258	−32.87
	Quercetin	−5.86	n.f*	n.f*	ILE4, VAL3, ALA122, TYR26, LEU29, ILE80, GLY81, LEU82, VAL84, ASP6, THR5, ACE2, GLU28, THR27, GLU125, THR85, ASP83	−38.71
	Imipenem	−5.34	03	GLU268, GLU269, ASN250	ARG263, THR266, TRP270, ASP291, LEU290, PRO289, ALA245, PHE248, TYR249, LYS251	−36.96
	Cyclophosphamide	−4.12	01	GLU269	GLU268, TRP270, LEU290, PRO289, ALA245, PHE248, TYR249, ASN250, LYS251, ASP252	−20.46

Abbreviation: n.f*; not found.

**Table 2:** Phytochemical screening of extracts of *Carica papaya* leaves.

Phytoconstituents	Extracts of *Carica papaya* Leaves		
	Aqueous	Methanol	Ethanol
Alkaloid	+	++	+++
Flavonoids	++	+	+++
Phenolic compound	−	+	−
Terpenoids	−	−	+++
Saponins	−	+	++
Glycosides	+	−	+

+, least positive; ++, more positive; +++, most positive; −, negative.

**Table 3:** DPPH analysis of different extracts of *C. papaya* leaves.

Concentration (μg/mL)	Types of Extracts			
	Aqueous (%)	Methanol (%)	Ethanol (%)	Ascorbic Acid (%)
20	51.26 ± 1.47	70.43 ± 3.47	77.86 ± 3.08	7.23 ± 1.67
40	65.90 ± 1.41	75.10 ± 0.53	83.73 ± 5.28	15.40 ± 2.24
60	69.30 ± 1.26	80.66 ± 2.46	85.23 ± 3.93	37.46 ± 7.18
80	77.33 ± 1.31	85.23 ± 4.60	88.76 ± 1.35	56.16 ± 7.98
100	80.66 ± 0.09	89.00 ± 1.20	89.63 ± 1.73	60.76 ± 2.28

## Data Availability

Not applicable.
